# Preliminary experience in pulmonary metastatectomy

**DOI:** 10.1186/1749-8090-10-S1-A269

**Published:** 2015-12-16

**Authors:** Sameh I Sersar, Ibrahim M Yassin

**Affiliations:** 1 Cardiothoracic Surgery, Mansoura University, 35516, Egypt; 2 King Abdullah Medical City, Makkah 21955, Saudi Arabia; 3 Cardiac Surgery Department, Saud al-Babtin Cardiac Center (SBCC), Al-Dammam, 31463, KSA; 4 Cardiothoracic Surgery Department, Tanta University Hospitals, Tanta, 31111, Egypt

## Background/Introduction

The surgical resection of pulmonary metastasis is not new it dates back to 1994 thanks to Blalock who resected lung metastases from colorectal cancer. He reported his preliminary results in 1947. Since then, there is a wide variation in the 5-year survival rate achieved by lung metastatectomies in different institutions. It varies between 9% to 45%.

## Aims/Objectives

We aimed to review our preliminary limited experience in pulmonary metastatectomies in KAMC; Makkah; Saudi Arabia in more than 3 years.

## Method

This is a retrospective study including 21 patients who had pulmonary metastatectomy in KAMC; Makkah between 2012 January and June 2015.

## Results

Our study included 21 patients who had lung metastases and were operated upon by thoracic surgeons. Females constituted more than 2/3 of this series. Mean age was 26 years. Mean follow up duration was 25 months. The commonest primary originated from the bones and rectosigmoid. Thirty five thoracic surgeries were required for those 21 cases. Twelve patients had one thoracic surgery, five had 2 surgeries, three had 3 surgeries and one had 4 surgeries. We used either VATS or thoracotomy in our lung metastatectomies. Only one third of the resected specimens were proved to be metastatic. No significant perioperative complications were reported in our series. No mortality was reported during the study period.

## Discussion/Conclusion

Pulmonary metastatectomy can be performed safely either by VATS or through thoracotomy with acceptable results.

